# Ricin as a Biothreat Agent: From Molecular Mechanisms to Clinical Toxicology, Forensic Aspects, and Risk Mitigation

**DOI:** 10.1155/bmri/5594252

**Published:** 2026-05-16

**Authors:** Miroslav Pohanka

**Affiliations:** ^1^ Military Faculty of Medicine, University of Defence, Hradec Králové, Czech Republic, unob.cz

**Keywords:** biohazard, biological terrorism, biological warfare agent, biological weapon, castor bean, CBRN, *Ricinus communis*, toxin

## Abstract

Ricin, a potent ribosome‐inactivating protein derived from the seeds of *Ricinus communis* L., represents a significant threat in the context of biological warfare and terrorism due to its high toxicity, ease of extraction, and lack of a specific antidote. This review provides a comprehensive examination of ricin’s biochemical structure, toxicological mechanisms, and clinical manifestations, alongside its historical and potential use as a weapon. The paper explores the global distribution and utility of the castor bean plant, outlines the molecular basis of ricin’s cytotoxicity, and evaluates current medical and technological countermeasures. Furthermore, it assesses international legal frameworks and policy instruments aimed at mitigating the misuse of biological agents. Case studies of ricin‐related incidents underscore the toxin’s appeal to both state and nonstate actors, highlighting the need for enhanced surveillance, public awareness, and interdisciplinary preparedness. The findings emphasize the dual‐use dilemma posed by ricin and advocate for sustained international cooperation to address emerging biothreats.

## 1. Introduction

Biological warfare agents are microorganisms or toxins that are used to cause disease or death in a targeted organism that can be humans, animals, or plants [1]. These biological warfare agents include bacteria, viruses, fungi, and toxins, each capable of inflicting significant harm as a result of targeted delivery [2]. For example, bacteria like *Bacillus anthracis*, the causative agent of anthrax, and viruses like *Variola major virus*, the causative agent of smallpox, can be weaponized to spread rapidly and cause widespread illness. Even the eradicated smallpox has military relevance as it is kept in microbial collections and its genome was mapped [3, 4]. Toxins such as botulinum toxin, produced by the bacterium *Clostridium botulinum*, are also highly potent biological warfare agents able to cause severe paralysis or death. The use of biological warfare agents for military purposes or in an act of terror is considered a serious threat due to their potential to cause large‐scale casualties, disrupt societies, and create fear and panic [5, 6]. International laws and treaties aim to prevent the development, production, and use of these dangerous agents. The Biological Weapons Convention, which entered into force in 1975 with the full name “Convention on the Prohibition of the Development, Production and Stockpiling of Bacteriological (Biological) and Toxin Weapons and on Their Destruction,” is the most important treaty that legislatively prevents the use of biological warfare agents for military purposes [7]. Nevertheless, the risks of using a biological warfare agent for attack by a criminal group, nonstate actor, or failed state exist. The combination of low costs and high efficacy of an attack can be attractive for such attackers. The term for biological and chemical weapons, “poor man’s atomic bomb,” concisely characterizes the problem [8, 9].

Ricin belongs among easily accessible biological warfare agents, and its accessibility and ease of manufacturing make it an effective menace when used for military or terrorist purposes [10]. Compared to other biological warfare agents like *B. anthracis*, *C. botulinum*, and botulinum toxin, ricin is rated as a less lethal (Category B of biological warfare agents as discussed further) but still relevant biological warfare agent [11–14]. This paper is focused on providing a comprehensive overview of ricin that includes its biochemical properties, sources, and mechanisms of action to analyze the potential of ricin as a biological weapon. The review was primarily focused on recent literature, with emphasis on peer‐reviewed journal articles published within the last 5 years. Nevertheless, older key papers were also included when they remained relevant or when recent literature on a specific aspect was limited or unavailable. It discusses historical instances, potential scenarios of misuse, and the impact on public health and safety to evaluate current countermeasures against ricin misuse, review existing detection methods, medical treatments, and preventive strategies, and identify gaps in current countermeasures. The article also highlights areas where current strategies may be insufficient or lacking, proposes new strategies and technologies, and discusses policy and regulatory frameworks.

## 2. *Ricinus communis* Plant

**Figure 1:**
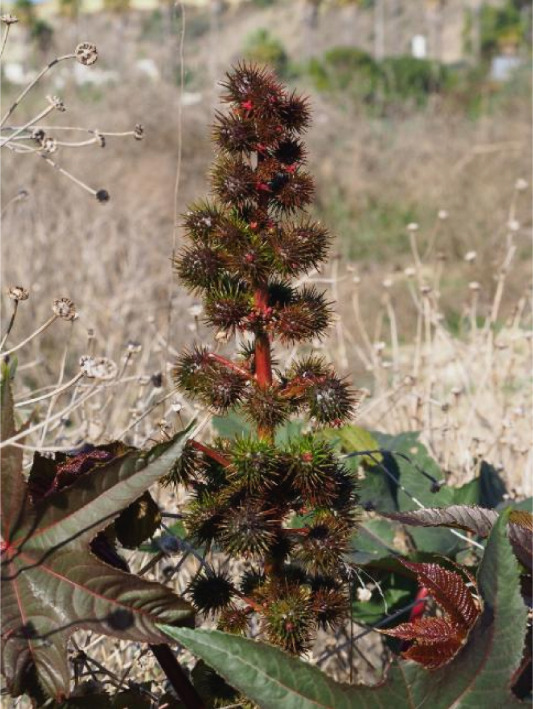
Castor bean (*Ricinus communis*) plant photographed by the author in southern Europe.

Ricin is a highly potent toxin derived from the seeds of the *R. communis* L. plant, commonly known as the castor bean plant. This plant is a member of the Euphorbiaceae family and is characterized by its large, glossy, palmate leaves and spiny seed capsules. The castor oil plant is native to the southeastern Mediterranean Basin, East Africa, and India, but it has become naturalized in tropical and subtropical regions worldwide [15–17]. *R. communis* thrives in warm climates and can grow as a perennial shrub or small tree, reaching heights even above 10 m in optimal conditions. The global distribution of *R. communis* is extensive due to its adaptability and the commercial value of castor oil. The plant’s ability to grow in diverse environments, from riverbanks to roadsides, has facilitated its spread across various continents. Biologically, *R. communis* exhibits a mixed pollination system, utilizing both wind and insects for pollination [18]. The plant’s resilience and rapid growth make it a valuable crop. A *R. communis* plant is depicted in Figure 1.

*R. communis* has a variety of practical applications that make it highly valuable. It is also approved to be a suitable plant for bioremediation of soil contaminated with heavy metals [19]. The seeds of this plant are the primary source of castor oil, which is widely used in the pharmaceutical and cosmetic industries [20, 21]. Some secondary metabolites of *R. communis* exert anti‐inflammatory and moisturizing properties [22–25]. The seeds of *R. communis* contain between 40% and 60% oil containing free fatty acids and are also rich in triglycerides, primarily ricinolein [26–28]. Castor oil is also utilized as a lubricant in machinery and as a raw material in the production of biodiesel, offering an eco‐friendly alternative to fossil fuels [29, 30]. Additionally, plant leaves can be used as a natural pesticide, helping to protect crops from pests without the need for synthetic chemicals. Despite its benefits, it is important to handle the plant with care because of its toxicity. Seeds harbor ricin, a water‐soluble protein that is known for its toxicity [31, 32]. Ricin inhibits protein synthesis in cells, leading to cell death and making it a potent biological weapon if misused.

In summary, the wide geographic distribution and robust growth of *R. communis*, together with its availability as an ornamental and industrial crop, mean that it can be planted and propagated with minimal effort. This practical accessibility constitutes an often‐overlooked security concern: individuals with malicious intent may readily obtain or cultivate the plant as a source of toxic material, which lowers the threshold for potential misuse. Therefore, awareness of *R. communis* cultivation and responsible handling of its seeds should be considered part of broader measures aimed at reducing ricin‐related risks.

## 3. Ricin Toxicity and Clinical Manifestations of Poisoning

**Figure 2:**
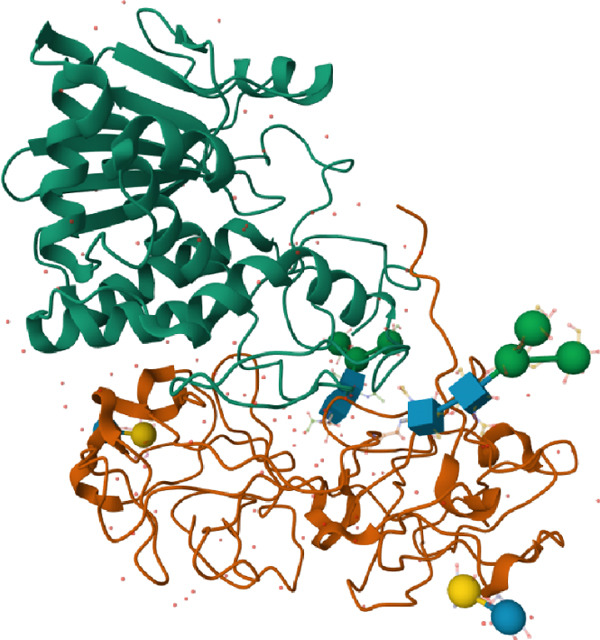
Ricin structure according to Rutenber et al. [35] depicted using the PDB database [36].

Ricin is a Type II ribosome‐inactivating protein (RIP), structurally consisting of two polypeptide subunits: the A chain (RTA) and the B chain (RTB), together giving a final heterodimer [33, 34]. The structure of ricin made by Rutenber et al. [35] is depicted using the PDB database [36] in Figure 2. The molecular weight of the RTA is 29 kDa, and the RTB has a similar weight of 28 kDa [37,38]. The molecular weights of the A and B subunits can vary across different journal articles due to posttranslational modifications, particularly glycosylation, which adds carbohydrate groups and increases the overall mass. Reported molecular weights typically range around 32 kDa for the A subunit and approximately 34 kDa for the B subunit, although these values may differ depending on the degree and type of glycosylation [39, 40]. Notably, ricin belongs to a broader class of RIPs, which share a conserved N‐glycosidase domain responsible for their cytotoxic mechanism [41–43]. Abrin, derived from *Abrus precatorius*, exhibits a homologous structure and comparable potency, targeting the same sarcin/ricin loop (SRL) site with similar enzymatic precision [44]. Despite differences in their B‐chain lectin domains, which influence cellular uptake and trafficking, both toxins converge mechanistically at the ribosomal level, underscoring a conserved strategy among Type II RIPs for translational arrest and cytotoxicity [45].

The RTA, which is enzymatically active, contains 267 amino acids and is responsible for the toxic effects of ricin [46, 47]. It functions by depurinating a specific adenine residue from the 28S rRNA of the ribosome, thereby halting protein synthesis, and it belongs to the rRNA N‐glycosylase (EC 3.2.2.22) family of enzymes [43, 48]. The RTA, following initial binding and internalization of the holotoxin, is trafficked via retrograde transport from the plasma membrane, or an early endosomal compartment, through the endosomal system to the trans‐Golgi network [49–52]. Subsequently, RTA progresses to the endoplasmic reticulum lumen. Upon reaching the endoplasmic reticulum, RTA must undergo reductive cleavage of the disulfide bond linking it to the RTB, a process often catalyzed by protein disulfide isomerase (PDI). The now‐free RTA then engages with the SEC61 translocon complex, typically recognized as a misfolded protein by the endoplasmic reticulum–associated degradation (ERAD) machinery, and utilizes this channel to be translocated (or retrotranslocated) into the cytosol. Once in the cytoplasm, the RTA protein folds into its active conformation as an N‐glycosidase to target the 28S rRNA of the 60S ribosomal subunit. Its catalytic activity by depurinating a specific adenine residue within the SRL of the 28S rRNA is responsible for toxicokinetics [53]. The SRL is a highly conserved structural element that constitutes a critical component of the GTPase‐Associated Center (GAC), which serves as the interaction platform for translational GTPases such as elongation factors EF‐G and EF‐Tu. This depurination event disrupts the functional integrity of the ribosome, effectively halting protein synthesis by impairing GTPase‐dependent translocation and factor binding.

The RTB, composed of 262 amino acids, is a lectin that binds to cell surface carbohydrates, facilitating the entry of the toxin into the cell [54, 55]. These chains are linked by a disulfide bond, which must be cleaved for the RTA to exert its toxic effects [56, 57]. The RTB’s structure is characterized by a *β*‐trefoil fold, typical of lectins, while the RTA contains both parallel and antiparallel *β*‐sheets alongside *α*‐helices [58,59]. This intricate structure allows ricin to effectively bind to and enter cells, making it a highly efficient toxin.

Considering ricin countermeasures, timely analysis of ricin in biological samples is a crucial task. Standard detection and quantification of ricin in complex matrices rely on a combination of immunological and analytical techniques, each offering distinct advantages in sensitivity and specificity. Immunoassays such as ELISA (enzyme‐linked immunosorbent assay) and lateral flow tests are widely employed for rapid screening, leveraging high‐affinity antibodies to recognize ricin epitopes with minimal sample preparation [60–63]. These methods are particularly valuable in field applications and preliminary diagnostics due to their portability and ease of use. For confirmatory analysis and structural characterization, mass spectrometry provides a robust platform capable of distinguishing ricin isoforms and detecting posttranslational modifications [64–67]. Coupling liquid chromatography with tandem mass spectrometry enhances resolution and allows for precise quantification even in trace concentrations. Integration of these methodologies into multiplexed platforms is advancing the reliability and throughput of ricin surveillance in both clinical and environmental contexts. There are also developed new tests and assays suitable for ricin surveillance [68, 69].

The mechanism of ricin toxicity is primarily based on its ability to inactivate ribosomes, which are essential for protein synthesis, consequently leading to cell death [70]. Upon entry into the cell, the disulfide bond between the RTA and RTB is reduced, allowing the RTA to reach the cytoplasm [71]. Once in the cytoplasm, the RTA enzymatically removes an adenine residue from the SRL of the 28S rRNA, a critical component of the ribosome [72, 73]. This depurination event halts protein synthesis, leading to cell death. Ricin’s high toxicity is attributed to its rapid inactivation of ribosomes, with the potential to inactivate up to 1500 ribosomes per minute [74]. The RTB’s lectin properties enhance the delivery of the RTA into the cell, ensuring that the toxin reaches its target efficiently. This dual‐chain mechanism, involving both binding and enzymatic activity, underscores the potency of ricin as a cytotoxin.

Clinical manifestations of ricin poisoning vary significantly depending on the route of entry, which includes ingestion, inhalation, and injection. Ingestion of ricin, often through contaminated food or the whole beans, leads to severe gastrointestinal symptoms such as nausea, vomiting, diarrhea, and abdominal pain, typically appearing within hours of exposure [75]. These symptoms can progress to more serious conditions like hemorrhagic gastroenteritis and multiorgan failure. Inhalation of ricin, which can occur through aerosolized particles, results in respiratory distress, including coughing, dyspnea, and pulmonary edema, often accompanied by fever and chest tightness. Injections, though less common, cause localized pain, swelling, and necrosis at the injection site, followed by systemic symptoms such as fever, headache, and hypotension. Ricin can also cause systemic toxicity through mucosal absorption, leading to widespread organ damage and potentially fatal outcomes. The damage to the heart, spleen, and bone marrow was, for instance, described in an experiment where ricin was intramuscularly administered to mice [76]. Median lethal doses (LD50) for ricin poisoning can significantly vary depending on the route of exposure. For instance, a median lethal dose for ricin of 1.01 *μ*g/kg when administered intraperitoneally and 28.3 mg/kg when administered perorally into Swiss albino male mice was described in a study by Kumar et al. [77]. In another study, LD50 of 1.7 *μ*g/kg was reported for dogs exposed to intravenously administered ricin [78]. LD50 for aerosolized ricin was calculated to be 5.8 *μ*g/kg for macaques [79]. LD50 for mice is 10 *μ*g/kg for intraperitoneal administration [80]. The expected LD50 for humans is around 5–10 *μ*g/kg for injection or inhalation administration [81]. The toxicity of ricin is quite high. Median lethal doses of other poisonous substances known from military use can be given for comparison. LD50 of nerve agent sarin given intramuscularly to rats is about 80 *μ*g/kg [82]. Hydrogen cyanide given to swine by inhalation leads to an LD50 of around 2.2 mg/kg [83]. Sulfur mustard has an LD50 value for rats poisoned intravenously equal to approximately 3 mg/kg [84]. Compared to these substances, ricin is more venomous. On the other hand, botulinum toxin reaches significantly lower values of LD50. The LD50 of botulinum toxin for humans is expected to be between 1 and 3 ng/kg for inhalation botulism [85]. Comparison of LD50 values is given in Table 1.

**Table 1:** Comparison of LD50 values for ricin and other selected warfare agents.

Warfare agent	LD50 value	Organism and type of administration	References
Botulinum toxin	1–3 ng/kg	Human, inhalation (estimated value)	[85]
Hydrogen cyanide	2.2 mg/kg	Swine, inhalation	[83]
Ricin	5.8 *μ*g/kg	Macaque, inhalation	[79]
Ricin	10 *μ*g/kg	Mouse, intraperitoneal	[80]
Ricin	1.01 *μ*g/kg/28.3 mg/kg	Intraperitoneal/peroral, albino Swiss mice	[77]
Ricin	1.7 *μ*g/kg	Dog, intravenous	[78]
Sarin	80 *μ*g/kg	Rat, intramuscular	[82]
Sulfur mustard	3 mg/kg	Rat, intravenous	[84]

Ricin toxicity can be positively used in medical applications, although it is a rare and still experimental approach. Ricin has garnered attention in oncological research due to its potent cytotoxicity, which can be harnessed for targeted cancer therapy when appropriately modified. Conjugation of RTA to monoclonal antibodies (mAbs) or other tumor‐specific ligands enables selective delivery to malignant cells, minimizing off‐target effects [86, 87]. This targeted approach facilitates intracellular inhibition of ribosomal function, leading to apoptosis in neoplastic tissues. Recent advancements in immunotoxin engineering have improved ricin’s therapeutic index by enhancing tumor selectivity and reducing systemic toxicity [88, 89]. Furthermore, encapsulation strategies and site‐specific delivery systems are being explored to optimize ricin‐based therapeutics for solid tumors and hematological malignancies [90, 91]. The facts about ricin structure and toxicity are summarized in Table 2.

**Table 2:** Survey of facts about ricin structure and toxicity.

Specification	Description or value	References
Type of molecule and basic structure	Heterodimeric protein composed of two subunits: A and B chains (RTA and RTB)	[33, 34]
Molecular weight	RTA: 29 kDa, RTB: 28 kDa, or glycosylated RTA around 32 kDa, RTB around 34 kDa	[37–40]
Role of RTA	Enzymatically removes an adenine residue from the sarcin/ricin loop of the 28S rRNA of the ribosome	[72, 73]
Role of RTB	Lectin that binds to cell surface carbohydrates, facilitating the entry of the toxin into the cell	[54, 55]
Mechanism of toxicity	Cytotoxicity through inactivation of ribosomes	[70]
Clinical manifestations	Nausea, vomiting, diarrhea, and abdominal pain, typically appearing within hours of exposure	[75]
LD50	5–10 *μ*g/kg for injection or inhalation administration to humans	[81]

From a critical perspective, the present section illustrates that ricin should be regarded as one of the highly dangerous toxic substances, even though several other biological toxins can exceed ricin in terms of acute potency under defined exposure conditions. This relative ranking, however, does not reduce the practical threat posed by ricin: the toxin can enter the body through multiple routes, and, once internalized, its ribosome‐inactivating activity may trigger severe systemic injury culminating in fatal outcomes. Therefore, the key message is not only that ricin is “less lethal” than some benchmark agents but also that its intrinsic toxicity combined with realistic routes of exposure makes it capable of causing clinically significant, and sometimes irreversible, poisoning.

## 4. Treatment of Ricin Poisoning

Treatment of ricin poisoning primarily focuses on supportive care, as there is no specific antidote available. Immunization by vaccines or administration of antibodies against ricin can be effective, and some studies have recommended such therapy based on outcomes from preclinical tests [92–97]. Immediate medical intervention is crucial to manage symptoms and prevent complications. For ingestion cases, activated charcoal may be administered to limit absorption, and gastric lavage can be performed to remove the toxin from the stomach [98]. Intravenous fluids are essential to maintain hydration and support renal function, while medications may be used to stabilize blood pressure and heart rate [99, 100]. In cases of inhalation, respiratory support, including oxygen therapy and mechanical ventilation, may be necessary to manage pulmonary symptoms. For injection‐related poisoning, wound care and systemic support are vital. Advanced treatments may involve the use of immunotherapy and research into potential antidotes, but these are still under development. Overall, the management of ricin poisoning requires a multidisciplinary approach to address the diverse and severe manifestations of this potent toxin.

In the absence of approved postexposure therapeutics for ricin intoxication, significant research has focused on immunological countermeasures that either prevent or neutralize the toxin before it exerts its cytotoxic effects. These strategies include recombinant subunit vaccines, mAb therapies, and rational epitope mapping to guide immunogen design and therapeutic antibody selection [101–105].

Among vaccine candidates, RiVax and RVEc have emerged as leading contenders [106–108]. RiVax is a recombinant protein vaccine derived from a mutated ricin RTA in which enzymatic activity is abrogated through site‐directed mutagenesis, preserving immunogenicity while eliminating toxicity. It has demonstrated protective efficacy in murine and nonhuman primate models against both parenteral and aerosolized ricin challenge. RVEc, developed using a similar approach, incorporates additional structural refinements to enhance stability and expression yield in eukaryotic systems. Both vaccines elicit robust humoral responses, with neutralizing IgG titers correlating with protection. Importantly, adjuvant formulation and thermostabilization have been optimized to support stockpiling and deployment in biodefense scenarios.

Passive immunotherapy using mAbs offers an alternative or complementary approach, particularly for postexposure treatment. Numerous mAbs targeting distinct epitopes on RTA and RTB have been characterized, with several demonstrating potent neutralizing activity in vitro and in vivo [109–111]. These antibodies function by blocking ricin’s binding to cell surface galactose residues, inhibiting endocytosis, or interfering with intracellular trafficking and enzymatic activity. Cocktail formulations combining mAbs against both subunits have shown synergistic effects, extended therapeutic windows and improved survival outcomes in animal models.

A critical component of both vaccine and antibody development is the precise mapping of neutralizing epitopes. High‐resolution structural studies, including X‐ray crystallography and cryo‐electron microscopy, have identified conformational epitopes on RTA’s enzymatic cleft and RTB’s carbohydrate recognition domains [112–116]. Epitope mapping using peptide arrays, mutagenesis, and competition assays has further delineated immunodominant regions and informed rational immunogen design. These efforts have enabled the engineering of epitope‐focused vaccines and the selection of mAbs with optimal binding kinetics and neutralization profiles.

Collectively, these immunological strategies represent a multifaceted defense against ricin poisoning. While no countermeasure has yet received regulatory approval, the translational progress of RiVax, RVEc, and mAb cocktails underscores the feasibility of both prophylactic and therapeutic interventions. Continued refinement of epitope‐targeted approaches and integration with rapid diagnostic platforms may ultimately enable effective clinical management of ricin exposure in both civilian and military contexts.

## 5. International Legislative Framework

The post–World War I period saw significant efforts to establish international peace and security, culminating in the formation of the League of Nations in 1919. One of the earliest treaties addressing biological warfare was the “Protocol for the Prohibition of the Use in War of Asphyxiating, Poisonous or Other Gases, and of Bacteriological Methods of Warfare,” commonly known as the “Geneva Protocol of 1925,” signed on June 17, 1925 [117, 118]. This protocol prohibited the use of chemical and biological weapons in war. Although it did not ban the possession or development of these weapons, it marked a crucial step toward international regulation of biological warfare, reflecting the global community’s growing awareness of the devastating potential of such weapons.

Following the devastation of World War II, the international community sought to strengthen disarmament efforts. This led to the creation of the “Convention on the Prohibition of the Development, Production and Stockpiling of Bacteriological (Biological) and Toxin Weapons and on Their Destruction,” known as the “Biological Weapons Convention” and signed on April 10, 1972 [119]. The Biological Weapons Convention, which entered into force on March 26, 1975, was the first multilateral treaty to ban the development, production, and stockpiling of biological and toxin weapons. It established a global norm against biological warfare, although its effectiveness has been limited by the absence of a formal verification regime. Despite these limitations, the Biological Weapons Convention represents a significant milestone in international efforts to prevent the proliferation of biological weapons.

The regulations about biological warfare were further developed by the United Nations, resulting in several resolutions and conventions. The “Convention on the Prohibition of Military or Any Other Hostile Use of Environmental Modification Techniques,” commonly known as the “Environmental Modification Convention” and signed on May 18, 1977, was created to prevent the use of environmental manipulation as a method of warfare [120]. This treaty prohibits the deliberate alteration of natural processes—such as weather, climate, or geological activity—when such actions are intended to cause widespread, long‐lasting, or severe effects. While not exclusively focused on biological warfare, the Environmental Modification Convention is relevant because it encompasses scenarios where the environment could be used to spread disease or disrupt ecosystems in a way that mimics biological attacks. The convention reflects a broader understanding of warfare, recognizing that environmental stability is a critical component of international peace and security.

The “United Nations Security Council Resolution 1540” was adopted unanimously on April 28, 2004, to address the growing concern that nonstate actors, particularly terrorist organizations, could acquire weapons of mass destruction, including biological weapons. This resolution obliges all United Nations member states to implement and enforce effective measures to prevent the proliferation of nuclear, chemical, and biological weapons, as well as their means of delivery. It requires states to establish domestic controls over related materials through legislation, law enforcement, and border security. Unlike traditional arms control treaties, Resolution 1540 is binding under Chapter VII of the United Nations Charter, giving it a unique legal force and global reach. It also encourages international cooperation and assistance to help countries build the necessary infrastructure to comply with its mandates.

The “International Health Regulations (2005)” established by the World Health Organization serve as a legally binding framework for 196 countries to detect, assess, report, and respond to public health threats, including those that may arise from biological incidents [121]. It entered into force on June 15, 2007. Although not a disarmament treaty, the International Health Regulations play a vital role in global biosecurity by ensuring that countries maintain core capacities to manage outbreaks, whether naturally occurring or deliberately caused. The regulations require prompt notification of events that may constitute a public health emergency of international concern, facilitating coordinated international responses. By strengthening surveillance and response systems, the International Health Regulations help mitigate the risks posed by biological weapons and enhance global resilience against pandemics and bioterrorism.

The effort to reduce the risk of use of a biological warfare agent followed even after the Biological Weapons Convention entered into force. The Australia Group, established in 1985, is an informal forum of countries that aims to prevent the proliferation of chemical and biological weapons through the harmonization of export controls [122]. It was established as a reaction to the use of chemical weapons during the Iran–Iraq War (the First Gulf War). All members of the Australia Group are parties to the Biological Weapons Convention and actively participate in efforts to strengthen the treaty. The group’s activities support the objectives of the Biological Weapons Convention by enhancing the effectiveness of national export licensing measures. By coordinating their export controls, member countries work to ensure that exports do not contribute to the development of chemical or biological weapons, thereby supporting global nonproliferation efforts.

Biological warfare involves the use of pathogens or toxins to harm or kill humans, animals, or plants during conflict. Bacteria such as *B. anthracis* and *Yersinia pestis* have been employed as biological weapons due to their ability to cause widespread illness. Viruses like variola virus (the cause of smallpox) and toxins such as botulinum toxin, staphylococcal enterotoxin B, and ricin are also considered potent biological agents [123–125]. The regulation of these agents is crucial to prevent their misuse and ensure public safety, and the aforementioned regulations are relevant to ricin. International treaties and conventions play a vital role in establishing norms and guidelines for the handling and control of these dangerous substances, thereby reducing the risk of their use in warfare.

Recent advancements in biotechnology have raised concerns about the potential for new and more effective biological weapons. Innovations in genetic engineering and artificial intelligence are being explored to enhance detection and response capabilities. Despite these advancements, the threat of biological warfare remains significant, necessitating ongoing international cooperation and robust regulatory frameworks. The continuous evolution of technology underscores the importance of adapting and updating international treaties to address emerging threats and ensure global security. Basic international regulations on biological warfare agents are summarized in Table 3.

**Table 3:** Basic international regulations and associations on biological warfare agents.

Treaty, protocol, or resolution	Organizing institution	Starting date
Protocol for the Prohibition of the Use in War of Asphyxiating, Poisonous or Other Gases, and of Bacteriological Methods of Warfare	League of Nations	Signed on June 17, 1925
Convention on the Prohibition of the Development, Production and Stockpiling of Bacteriological (Biological) and Toxin Weapons and on Their Destruction	United Nations	Signed April 10, 1972, entered into force on March 26, 1975
Convention on the Prohibition of Military or Any Other Hostile Use of Environmental Modification Techniques	United Nations	Signed on May 18, 1977
United Nations Security Council Resolution 1540	United Nations	Adopted on April 28, 2004
International Health Regulations (2005)	World Health Organization	Entered into force on June 15, 2007
The Australia Group	Informal forum of countries	Established in 1985

In practical terms, these international instruments also shape how states address the criminal misuse of ricin. Ricin is treated in international law as a toxic biological agent, and its development, acquisition, stockpiling, transfer, and use for hostile purposes are prohibited or strictly controlled through binding conventions and related United Nations measures. By codifying toxins within the broader category of biological weapons, these frameworks require national implementation (e.g., criminal offenses, export and precursor controls, and investigative powers) so that ricin is not only regulated merely as a hazardous chemical but also legally approached as a biological warfare agent with corresponding penal and security consequences.

## 6. Potential Use of Ricin in the Military and Bioterrorism

Ricin stands out as a potent biological weapon due to its high toxicity, ease of concealment, and lack of a known antidote. Even in minuscule quantities, it can cause death within days if inhaled, ingested, or injected. Its lethality, combined with the difficulty of early detection and treatment, makes it a formidable agent in asymmetric warfare and terrorism [126–128]. Unlike many chemical or radiological threats, ricin does not require sophisticated delivery systems to be effective, which increases its appeal to nonstate actors and lone perpetrators seeking to inflict mass harm or incite panic.

In a bioterrorism context, isolating ricin from castor beans involves a sequence of relatively simple yet effective biochemical steps that can be carried out with minimal equipment. The process typically begins with the mechanical dehusking and grinding of castor seeds into a fine mash, which releases the oil and protein content [129]. The mash is then subjected to aqueous extraction, where it is mixed with water or a buffered solution to dissolve the water‐soluble ricin protein. After stirring and allowing sufficient time for solubilization, the mixture is filtered to remove insoluble plant debris, leaving behind a crude protein‐rich solution [130].

To concentrate the ricin, the solution may undergo ammonium sulfate precipitation, a common protein purification technique that exploits differences in solubility [131, 132]. By gradually adding the salt, ricin can be selectively precipitated out of the solution while other proteins remain dissolved. The precipitate is then collected by centrifugation and redissolved in a smaller volume of buffer. Further purification can be achieved using dialysis to remove excess salts or through chromatographic methods such as ion exchange or gel filtration, which separate proteins based on charge or size [132–134]. While these latter steps require more technical knowledge, they are not beyond the reach of individuals with basic laboratory training.

Importantly, none of these procedures require access to restricted chemicals or advanced laboratory infrastructure. Many of the necessary materials, such as centrifuges, filters, and reagents, can be sourced from educational suppliers or improvised using household items. Online forums and open‐access scientific literature have, unfortunately, made this knowledge more accessible, lowering the barrier for malicious use. The relative ease of isolating ricin, combined with the difficulty of detecting small‐scale production, underscores the importance of monitoring precursor materials and educating the public about the risks associated with castor bean misuse.

From a military standpoint, ricin has limited strategic utility compared to other biological agents due to its relatively slow onset and noncontagious nature. However, its psychological impact and potential for targeted assassinations or sabotage operations make it valuable in unconventional warfare [135, 136]. Ricin can be weaponized in aerosol form or incorporated into munitions for localized effects, particularly in scenarios where stealth and deniability are prioritized over mass casualties. Its use is more aligned with covert operations than with battlefield deployment, reflecting its niche role in military arsenals.

The Centers for Disease Control and Prevention classify biological warfare agents into Categories A, B, and C based on their threat level, ease of dissemination, and potential impact on public health [137–140]. Ricin is placed in Category B, which includes agents that are moderately easy to disseminate and result in moderate morbidity but generally low mortality [141–143]. This classification reflects ricin’s unique position: while it is highly toxic and capable of causing death, it lacks the contagious nature and mass‐casualty potential of Category A agents like *B. anthracis* (the causative agent of anthrax), *Y. pestis* (the causative agent of plague), *C. botulinum* with produced botulinum toxin, or *Variola major virus* (the causative agent of smallpox). Unlike Category C agents, which are considered emerging threats with potential for future weaponization, ricin is already well understood and has a documented history of use in targeted attacks. Its placement in Category B underscores a dual reality—it is not the most catastrophic agent in terms of scale, but its accessibility, psychological impact, and potential for covert deployment make it a persistent and credible threat in the realm of bioterrorism.

One of the strategic limitations of ricin as a military‐grade biological weapon lies in the delayed timeline required for its production. Unlike synthetic agents that can be manufactured on demand, ricin depends on the cultivation of castor bean plants, which introduces a significant lag between planning and deployment. Military operations that require rapid readiness or immediate scalability are hindered by the agricultural phase, as castor plants must reach maturity before their seeds can be harvested and processed. This agricultural dependency reduces ricin’s viability for fast‐response scenarios and makes its production more predictable and potentially detectable during the early stages of weaponization.

**Figure 3:**
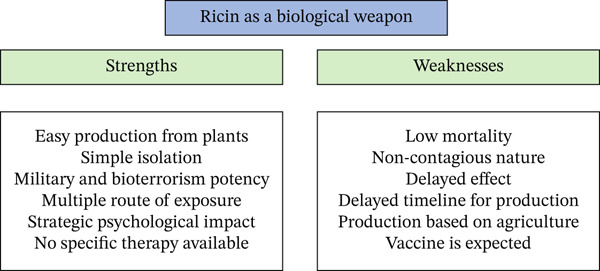
Biological weapons with ricin: strengths and weaknesses.

In bioterrorism, ricin’s relevance lies in its symbolic and disruptive potential rather than its capacity for mass destruction. Its use in letters, public spaces, or food and water supplies can generate widespread fear and media attention, amplifying its psychological impact far beyond the number of actual victims. The accessibility of raw materials and the low technological barrier to weaponization make it a persistent concern for counterterrorism agencies. Ricin exemplifies the kind of low‐cost, high‐impact threat that modern security frameworks must continuously adapt to detect and neutralize. The strengths and weaknesses of ricin as a biological weapon are depicted in Figure 3.

Against this background, ricin may be viewed as a kind of “weapon of the poor”: while it is not among the most militarily effective toxicants, it remains comparatively accessible and usable at a small scale by actors who cannot obtain more efficient agents or lack the technical capacity for sophisticated production and dissemination. This dynamic is particularly relevant in asymmetric conflicts, where weaker protagonists may seek attainable means to impose fear, disruption, or selective casualties. Health service agencies and other organizations tasked with protection against biological warfare agents should therefore treat ricin not as a marginal hazard but as an additional, realistic risk that warrants explicit inclusion in preparedness planning, surveillance, and interagency coordination.

## 7. Lessons Learned From Ricin Cases

The analysis of documented ricin‐related incidents provides critical insights into the threat posed by this potent toxin and informs strategies for both prevention and response. Case studies involving intentional ricin dissemination highlight the diverse methods of delivery and underscore the importance of early detection systems, public health preparedness, and interagency coordination. These cases reveal common vulnerabilities, such as delays in diagnosis due to nonspecific symptoms and challenges in forensic attribution. By systematically examining these events, researchers and policymakers can identify patterns in attacker behavior, improve biosurveillance infrastructure, and develop targeted countermeasures, including public awareness campaigns, rapid diagnostic tools, and stockpiling of supportive medical treatments. Ultimately, such case‐based learning enhances our capacity to mitigate the impact of future ricin attacks and strengthens biodefense resilience.

On September 7, 1978, Georgi Markov, a Bulgarian émigré and journalist, was attacked in London, United Kingdom, while waiting at a bus stop near Waterloo Bridge [144]. The assailant, believed to be an agent of the Bulgarian secret services operating with Soviet support, used a modified umbrella to surreptitiously implant a 1.7‐mm‐diameter pellet with ricin into Markov’s right thigh. The pellet, composed of a platinum–iridium alloy, was engineered with two microscopic holes sealed with a sugar‐based substance identified as ricin in the later investigation. The pellet facilitated delayed release of a toxic agent [145–147]. Markov developed acute febrile symptoms within hours and died 4 days later in St. James’s Hospital. A forensic investigation, including radiographic imaging and autopsy, identified the foreign object and its sophisticated design, prompting international scrutiny and marking the case as a prototypical example of state‐sponsored clandestine assassination during the Cold War.

There were also notable criminal cases where ricin played a significant role, but these cases were not fully studied by scholars, and there is only minor information from journal articles [148, 149]. A case happened in Cologne, Germany, in 2018 when police authorities arrested Sief Allah H., a Tunisian national, who had successfully produced ricin from beans and was planning a terrorist attack. This was the first confirmed case of a jihadi terrorist in the West manufacturing ricin. The case underscored the role of internet surveillance and intelligence sharing in intercepting bioterror plots before execution. In another case from 2003, British police arrested several individuals suspected of preparing a ricin‐based attack on the London Underground. Although no ricin was ultimately recovered, the case showed that a scenario with a ricin attack would be a real threat and that the culprits had the knowledge and intention to create a ricin weapon. Another case happened in 2024 when Jason Kale Clampit, a tree cutter from Arkansas, was convicted of producing ricin in his home. While his motives remain unclear, it was suggested during the court procedure that he focused on setting traps for trespassers. The perpetrator was himself poisoned during manipulation with ricin. These cases collectively highlight the diverse motivations and methods behind ricin‐related incidents, reinforcing the importance of multidisciplinary approaches, including law enforcement, public health, and intelligence, to detect, deter, and respond to bioterror threats.

While ricin is widely recognized for its potential use in bioterrorism, it has also been implicated in cases of suicide, revealing a lesser‐known but equally concerning dimension of its misuse. Several individuals have attempted or committed suicide by ingesting castor beans, the natural source of ricin, often after self‐directed research into its toxic properties. In a study published in 2009, a 49‐year‐old man in Belgium committed suicide by injection of castor bean extract [150]. The patient presented symptoms such as nausea, vomiting, diarrhea, dyspnea, vertigo, and muscular pain. He died of multiorgan failure 9 h later despite symptomatic and supportive intensive care. In another case described in 2025, a 19‐year‐old patient ingested approximately 50 castor beans and was hospitalized [151]. Because of timely help, endoscopic removal of the seeds and application of charcoal, the patient survived without sequelae. A suicide attempt was also described by de Haan et al. [152] in a study published in 2016. They treated a 77‐year‐old woman who voluntarily took 15 crushed castor beans. The poisoned woman suffered from vomiting and diarrhea combined with comorbidities from other pathologies. The ricin poisoning was resolved by symptomatic treatment, and the woman was further transferred to the psychiatric department. A tragic report was published by Hoizey et al. [153] in 2016. They reported a suicide attempt by a 23‐year‐old man. He was self‐poisoned by a castor bean extract taken simultaneously orally and intravenously. Though he survived the poisoning, he succumbed to another suicidal attempt 25 h after admission when he threw himself out of a window. These incidents underscore the accessibility of castor beans and the misconception that natural origin equates to safety. They also highlight the need for public education on the dangers of plant‐derived toxins and the importance of mental health interventions to prevent such self‐harm scenarios. The cases mentioned above are summarized in Table 4.

**Table 4:** Summary of basic facts from the discussed cases.

Case	Year	Location	Type/context	Delivery/exposure	Outcome and keynote
Georgi Markov	1978	London, United Kingdom	State‐sponsored clandestine assassination (Cold War)	Ricin pellet (1.7 mm diameter) implanted in the right thigh using a modified umbrella	Developed acute febrile symptoms within hours; died 4 days later; forensic imaging/autopsy identified pellet design and ricin
Cologne ricin plot (Sief Allah H.)	2018	Cologne, Germany	Terrorism planning; ricin production from castor beans	Ricin produced from beans	Arrested before attack; highlighted the importance of internet surveillance and intelligence sharing
London Underground plot (suspected)	2003	London, United Kingdom	Suspected preparation of a ricin‐based attack	Planned ricin weapon (no ricin recovered)	Arrests made; no ricin ultimately recovered; illustrated the credibility of the ricin attack scenario
Jason Kale Clampit	2024	Arkansas, United States	Criminal case; home production	Produced ricin at home (reported intention: traps for trespassers)	Convicted; perpetrator reportedly poisoned himself during handling/manipulation
Adult male (49 years)	2009 (study published)	Belgium	Suicide	Injection of castor bean extract	Symptoms included nausea, vomiting, diarrhea, dyspnea, vertigo, and muscular pain; died of multiorgan failure 9 h later despite intensive supportive care
Patient (19 years)	2025 (study published)	Not specified	Suicide attempt/self‐poisoning	Ingestion of ~50 castor beans	Hospitalized; survived without sequelae due to timely care (endoscopic removal + charcoal)
Woman (77 years)	2016 (study published)	Not specified	Suicide attempt/self‐harm	Ingestion of 15 crushed castor beans	Vomiting and diarrhea plus comorbidities; poisoning resolved by symptomatic treatment; transferred to the psychiatric department
Adult male (23 years)	2016 (study published)	Not specified	Suicide attempt involving ricin preparation	Castor bean extract taken orally and intravenously	Survived poisoning but died 25 hours after admission due to another suicide attempt (jumped from a window)

Taken together, these examples support a critical conclusion: ricin‐related incidents span state‐sponsored assassination, terrorism planning, criminal experimentation, and self‐harm, and they therefore cannot be dismissed as rare historical anomalies. Across the cases, several recurring patterns are evident. First, ricin benefits from a uniquely low threshold for acquisition because the source material is widespread and legal in many settings, while the technical steps needed for crude preparation are within reach of individuals with limited resources. Second, early recognition is difficult because clinical manifestations are often nonspecific and route‐dependent and because clinicians may not initially suspect toxin exposure; this can delay supportive treatment and complicate outbreak investigation. Third, even when the number of victims is limited, the operational and societal consequences can be disproportionate, including costly emergency responses, public anxiety, and significant investigative efforts to determine intent and attribution. Finally, the diversity of motivations, from ideological violence to opportunistic criminality and personal self‐harm, means that risk mitigation cannot rely on a single sector. A common recommendation emerging from these lessons is that ricin should be treated as a relevant and persistent biological threat: preparedness plans should explicitly include ricin in differential diagnosis and laboratory readiness, strengthen intelligence and law‐enforcement cooperation around precursor monitoring and online activity, and support continued development and deployment of rapid detection and medical countermeasures.

## 8. Ricin Assays

Analytical detection of ricin requires a tiered approach that balances rapid field screening with high‐fidelity laboratory confirmation. Initial presumptive identification typically employs immunochromatographic platforms, such as lateral flow assays. These devices utilize a sandwich format where gold‐labeled or fluorescently tagged antibodies capture the toxin, providing a qualitative or semiquantitative result within minutes. While modern fluorescence‐based lateral flow assays have pushed the limit of detection into the low nanogram‐per‐milliliter range, these methods remain susceptible to matrix interference and often lack the specificity required to distinguish ricin from its less toxic but highly homologous relative, *R. communis* agglutinin (RCA120), which shares over 80% sequence identity in its A and B chains [154–156].

For quantitative laboratory assessment, ELISA remains the benchmark for high‐throughput screening and toxin quantification [157, 158]. By leveraging mAbs directed at distinct epitopes of the RTA and RTB, ELISA provides the sensitivity necessary to detect trace amounts of the protein in complex environmental and biological matrices. However, a significant limitation of standard immunochemical methods is their inability to discern between biologically active toxin and denatured, nonfunctional protein. To address this, expert protocols often supplement ELISA with electrochemiluminescence for enhanced dynamic range or integrate functional assays [159, 160].

Unambiguous forensic confirmation is achieved through instrumental techniques, primarily liquid chromatography coupled with mass spectrometry or tandem mass spectrometry [161–164]. Using a bottom‐up proteomic strategy, the toxin is subjected to tryptic digestion to release signature peptides that serve as definitive molecular fingerprints. This approach facilitates the differentiation of ricin isoforms and the identification of cultivar‐specific variations that are beyond the resolution of immunological tools. To handle complex samples like food or serum, affinity enrichment, using either antibody‐functionalized magnetic beads or glycoprotein ligands like asialofetuin, is typically employed prior to mass spectrometric analysis. The current gold standard in toxicological forensics involves the correlation of these proteomic signatures with functional data from MALDI‐TOF mass spectrometry assays [165–168].

## 9. Conclusions

Ricin exemplifies the complex intersection of natural biochemistry and modern security challenges. Its accessibility, combined with potent cytotoxic effects and the absence of definitive treatment, positions it as a persistent concern in both military and civilian contexts. While its noncontagious nature limits its utility in large‐scale warfare, ricin’s psychological impact and potential for covert deployment make it a favored tool in asymmetric threats and targeted attacks. Historical incidents and recent criminal cases demonstrate that even rudimentary knowledge and equipment can suffice for weaponization, underscoring the importance of proactive risk mitigation. Although international treaties and regulatory frameworks provide a foundation for control, gaps remain in enforcement, detection, and public health readiness. Addressing these vulnerabilities requires a multidisciplinary approach that integrates scientific innovation, legal oversight, and global collaboration. In practice, ricin remains a biological risk because it combines a readily obtainable source material (castor beans) with a toxin that is effective at very low doses and difficult to recognize clinically in the early phase. This combination makes small‐scale incidents plausible and challenging to detect in time, especially when exposure occurs outside of well‐monitored settings. The current lack of an approved, widely available antidote means that outcomes depend strongly on early suspicion, rapid supportive care, and access to specialized diagnostics, which may not be uniformly available across healthcare systems. Therefore, preparedness should focus not only on high‐consequence mass‐casualty scenarios but also on the more likely spectrum of limited attacks, criminal misuse, and accidental exposures. Continued progress in rapid detection (including field‐deployable assays and confirmatory mass spectrometry), medical countermeasures (vaccines, neutralizing antibodies, and postexposure therapeutics), and forensic attribution tools is essential to reduce both health impacts and the broader societal disruption caused by fear and uncertainty. Finally, sustained attention to ricin highlights the wider dual‐use dilemma: advances that improve toxin research, biotechnology, and accessibility of information can simultaneously strengthen preparedness and lower barriers for misuse. As biotechnology advances, so too must our strategies for anticipating and countering the misuse of biological agents like ricin.

## Funding

The work was supported by the Ministry of Defence of the Czech Republic “Long Term Organization Development Plan” – Healthcare Challenges of WMD II of the Military Faculty of Medicine, Hradec Králové, University of Defence, Czech Republic (Project No. DZRO‐FVZ22‐ZHN II).

## Conflicts of Interest

The authors declare no conflicts of interest.

## Data Availability

All data are available within the article.
